# Sustainable Lifestyle Among Office Workers (the SOFIA Study): Protocol for a Cluster Randomized Controlled Trial

**DOI:** 10.2196/57777

**Published:** 2024-07-31

**Authors:** Katarina Bälter, Abby C King, Johanna Fritz, Annika Tillander, Oskar Halling Ullberg

**Affiliations:** 1 Division of Public Health School of Health, Care and Social Welfare Mälardalen University Västerås Sweden; 2 Department of Medical Epidemiology and Biostatistics Karolinska Institute Stockholm Sweden; 3 Stanford Prevention Research Center Department of Medicine Stanford University School of Medicine Stanford, CA United States; 4 Department of Epidemiology and Population Health Stanford University School of Medicine Stanford, CA United States; 5 Division of Physiotherapy School of Health, Care and Social Welfare Mälardalen University Västerås Sweden; 6 Department of Statistics and Machine Learning Linköping University Linköping Sweden

**Keywords:** diet, physical activity, work life, health promotion, climate change, sustainable lifestyle, citizen science, Our Voice

## Abstract

**Background:**

Society is facing multiple challenges, including lifestyle- and age-related diseases of major public health relevance, and this is of particular importance when the general population, as well as the workforce, is getting older. In addition, we are facing global climate change due to extensive emissions of greenhouse gases and negative environmental effects. A lifestyle that promotes healthy life choices as well as climate and environmentally friendly decisions is considered a sustainable lifestyle.

**Objective:**

This study aims to evaluate if providing information about a sustainable lifestyle encourages individuals to adopt more nutritious dietary habits and increase physical activity, as compared to receiving information solely centered around health-related recommendations for dietary intake and physical activity by the Nordic Nutrition Recommendations and the World Health Organization. Novel features of this study include the use of the workplace as an arena for health promotion, particularly among office workers—a group known to be often sedentary at work and making up 60% of all employees in Sweden.

**Methods:**

The Sustainable Office Intervention (SOFIA) study is a 2-arm, participant-blinded, cluster randomized controlled trial that includes a multilevel sustainable lifestyle arm (intervention arm, n=19) and a healthy lifestyle arm (control arm, n=14). The eligibility criteria were being aged 18-65 years and doing office work ≥20 hours per week. Both intervention arms are embedded in the theoretically based behavioral change wheel method. The intervention study runs for approximately 8 weeks and contains 6 workshops. The study focuses on individual behavior change as well as environmental and policy features at an organizational level to facilitate or hinder a sustainable lifestyle at work. Through implementing a citizen science methodology within the trial, the participants (citizen scientists) collect data using the Stanford Our Voice Discovery Tool app and are involved in analyzing the data, formulating a list of potential actions to bring about feasible changes in the workplace.

**Results:**

Participant recruitment and data collection began in August 2022. As of June 2024, a total of 37 participants have been recruited. The results of the pilot phase are expected to be published in 2024 or 2025.

**Conclusions:**

Given the ongoing climate change, negative environmental effects, and the global epidemic of metabolic diseases, a sustainable lifestyle among office workers holds important potential to help in counteracting this trend. Thus, there is an urgent unmet need to test the impact of a sustainable lifestyle on food intake, physical activity, and environmental and climate impacts in a worksite-based randomized controlled trial. This study protocol responds to a societal need by addressing multilevel aspects, including individual behavior changes as well as environmental and organizational changes of importance for the successful implementation of sustainable lifestyle habits in an office setting.

**International Registered Report Identifier (IRRID):**

DERR1-10.2196/57777

## Introduction

### Background

Nations around the world, including Sweden, are facing multiple challenges driven by lifestyle- and age-related diseases of major public health relevance, such as diabetes type 2, cardiovascular disease, and cancer [1,2]. It is estimated that physical inactivity in the European population costs each country between €1 and €2 billion (US $1.09 and US $2.18 billion) in health care spending [3]. Moreover, improved adherence to healthy dietary patterns by 20% in the American population has the potential to save more than US $20 billion in costs associated with noncommunicable diseases (NCDs) [4]. This is of particular importance when the general population, including the global workforce, is getting older. Moreover, we are facing global climate change due to extensive emissions of greenhouse gases and substantial negative environmental effects [5]. A lifestyle that promotes both healthy life choices, as well as climate- and environmental-friendly behaviors, is considered a sustainable lifestyle [6,7]. It is important that individuals believe that they can take responsibility for their own lifestyle, but it is also essential that a sustainable lifestyle be encouraged and facilitated at work and through society at large.

Although many Swedish workplaces offer their employees tax-deductible preventive health care services (wellness allowance), such incentives have been used mostly to promote exercise outside of the workplace and have been used predominantly by employees who are already physically active [8,9]. In order to achieve a paradigm shift in the way we think about lifestyle factors and health in relation to work, a sustainable lifestyle in areas such as physical activity and healthful food access needs to be considered when new offices are built and designed in order to encourage and facilitate an active and healthy lifestyle during working hours. Such changes need to be feasible, user-friendly, and attractive, but at the same time, avoid creating undue pressure or feelings of guilt among employees [10,11]. A previous study by our team with 5364 well-educated adults aged 18-45 years showed that participants consumed, on average, 1 meal with meat per day for lunch or dinner, and the carbon footprint from their food consumption was, on average, 4.7 kg greenhouse gas emissions per person per day (CO_2_ equivalents [CO_2_e]), which corresponds to 1.7 tons of greenhouse gas emissions per person per year [12]. As different food sources generate different amounts of carbon emissions, with plant foods generally having lower emissions than animal products [13], there would be substantial climate benefits if more people reduced their intake of beef and increased their intake of plant-based foods. Moreover, epidemiological studies have consistently found that high consumption of plant-based foods, such as fruits, vegetables, legumes, and whole-grain products, reduces the incidence of NCDs, such as obesity, diabetes type 2, cardiovascular diseases, and certain types of cancer [14-16]. Health-enhancing behaviors such as regular physical activity are also linked to reduced risk of NCDs [17]. One form of physical activity is active commuting, such as walking or biking to work, which also has positive climate and environmental dimensions as it does not contribute to greenhouse gas emissions, traffic congestion, air pollution, or noise [18,19]. The above types of behavior changes would result in more nutritious and climate-friendly food habits and increased physical activity, which would impact public health substantially.

### Current Guidelines

To promote good health and prevent major NCDs in the population, the Nordic countries have common dietary recommendations, the Nordic Nutritional Recommendations (NNR) [20,21], and Sweden has adopted the recommendation about physical activity from the World Health Organization (WHO) [22]. However, such preventive actions have consistently had a poor impact on the public’s lifestyle choices, and few community members are aware of these recommendations. Therefore, new ways of impacting people’s lifestyles are needed. Since 60% of all employees in Sweden work in an office setting [23], we suggest that a shift to healthy plant-based food should be encouraged at workplaces in the form of food served at events, catering, coffee breaks, and lunch, and physical activity should be encouraged by facilitating active commuting, walk-and-talk meetings, and other forms of everyday movement during office hours.

### Objective

The overall aim of the Sustainable Office Intervention (SOFIA) study is to promote and facilitate the adoption of a sustainable lifestyle within an office environment, with the dual goals of enhancing public health and mitigating the adverse effects of climate change and negative environmental impact. Specifically, the research aims to assess the effectiveness of promoting sustainable lifestyle choices in the workplace compared to promoting a conventional healthy lifestyle.

## Methods

### Study Design and Sample Size

**Figure 1:**
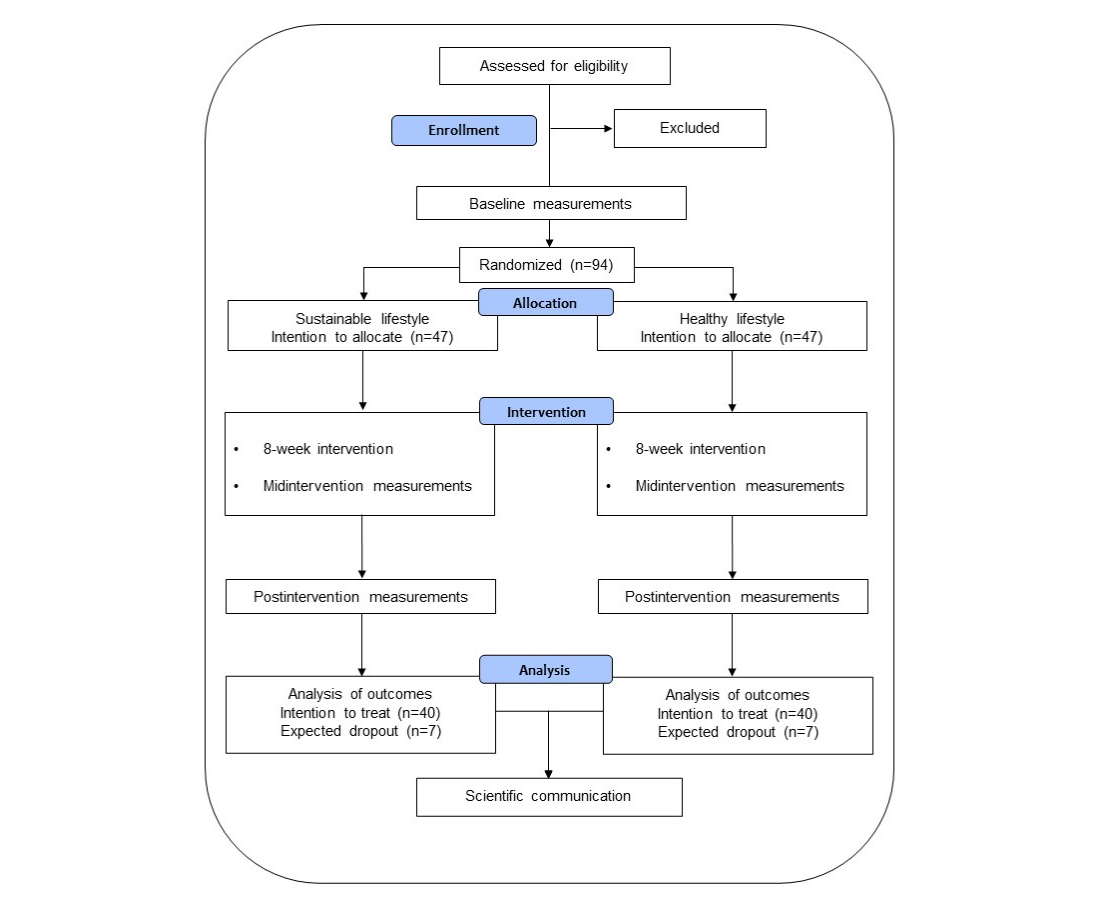
A SPIRIT flow chart of the SOFIA study. Based on power calculations from the study CLEAR 2018 (unpublished data), 94 participants will be recruited, accounting for an assumed dropout rate of 10% to 15% after the baseline assessment. SOFIA: Sustainable Office Intervention; SPIRIT: Standard Protocol Items: Recommendations for Interventional Trials.

**Figure 2:**
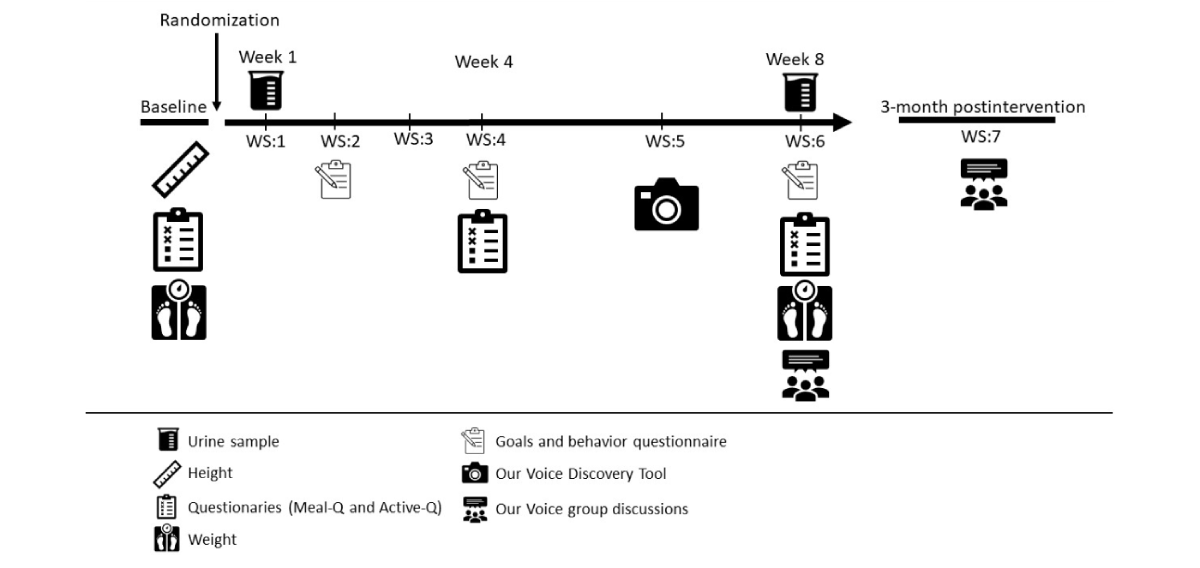
Timeline for the SOFIA 8-week intervention study, which includes 6 workshops plus a follow-up meeting occurring 2-3 months after week 8. WS: workshops. SOFIA: Sustainable Office Intervention.

The SOFIA study is part of a larger mixed-method effort titled *Concepts for the Sustainable Office of the Future* (SOFCO) [24], which is studying how sustainable lifestyles and health may be supported among office workers in Sweden. Here, we describe the SOFIA substudy, which is a 2-arm, single-blind (participants), cluster randomized controlled trial comparing a sustainable lifestyle intervention arm with a healthy lifestyle control arm (Figure 1). The intervention study, which will occur in office sites in the Mälardalen region, Sweden, runs over 8 weeks, with each intervention consisting of 6 workshops occurring every week for the first 4 weeks and then every second week for the last 4 weeks (Figure 2). The data collection will occur at baseline and after 4 and 8 weeks, with the target number of randomly assigned participants per intervention arm being 47 participants each.

### Ethical Considerations

The SOFIA study was approved by the Swedish Ethical Review Authority (2021-02309) concerning biological samples and research on human subjects. The study was registered at ClinicalTrials.gov as protocol ID 1-2020/0244. The research activities are conducted according to ethics principles set by the Declaration of Helsinki [25], and the study protocol strictly follows regulations by the Swedish Research Council concerning informed consent and confidentiality [26]. Trained personnel from the research team are responsible for data entry and cleaning. A senior statistician (AT) and data managers (OHU and KB) will ensure accurate and efficient data collection and analysis and guarantee confidentiality for the study participants. All personal data collected will be stored within the framework of European Data Protection Agreements [27] and on a 2-step authorized, password-protected server only accessed by authorized members of the research team. In addition, the Stanford Discovery Tool data are retained in a secure data repository that is reviewed annually by the Stanford University Institutional Review Board for Human Subjects Protections, as well as the Stanford Office of the General Counsel and the University Privacy Office, to ensure that it remains compliant with all European General Data Protection Regulations [27]. Prior to data collection, all participants will be provided with written and oral information about the study design and sign the informed consent form regarding all aspects of the study including primary and secondary objectives. Collected data will be deidentified using study-specific ID numbers. When the participants use the Discovery Tool to take photos, they will be instructed to avoid taking photos of persons or objects that may be linked to a specific person. The research team will review all photos, and if needed, blur humans or company logos to protect the confidentiality of participants. The participants will not be compensated, as participation takes place during working hours and participants are insured by their employer.

### Study Population

The project is being conducted in collaboration with companies in the office sector in Sweden. Company A owns and develops office buildings, is located in a medium-sized city, and houses approximately 35 office workers. Company B coordinates and develops facility management, is located in a large city, and has approximately 400 office workers. Through advertisement at these offices, we will invite office workers to sign up via a short web-based screening questionnaire to confirm eligibility for the study, the number of hours working in their office per week, the office location, and active commuting and food habits, along with their contact information. The questionnaire takes approximately 2-5 minutes to complete and will be administrated using links and QR codes. Eligible study participants are aged 18-65 years and perform office work ≥20 hours per week. Exclusion criteria comprise not having access to a smartphone compatible with the Discovery Tool (such as iPhone 5 or Android 2, 3, or later versions), being blue-collar workers who perform ≥20 hours per week of strength or physical work rather than office work, having mental disabilities, and not speaking Swedish.

### Procedure

Invitations to prospective participants working at the targeted companies will be sent through email, and upon their confirmation of interest, individuals will be subsequently invited to attend a baseline meeting. The primary objective of this meeting will be to provide individuals with comprehensive details about the study, have interested individuals sign the approved human subjects consent form, administer the baseline questionnaire, conduct anthropometrical assessments, and provide participants with a urinary sample test kit. A web-based questionnaire including questions about demographic factors, work environment, food habits, physical activity, workplace-related factors, as well as opinions and actions related to environmental and climate impact from one’s lifestyle, will be administered at baseline. The baseline questionnaire takes between 20 to 30 minutes to complete and will be delivered to the participants’ work email. A shorter version of the questionnaire, with a focus on food habits and physical activity, will be repeated at 4 and 8 weeks (Figure 2). The questions about food intake, diet-related CO_2_e, and physical activity have been evaluated with regard to validity and reliability [28-31]. Urinary samples will be collected at baseline and 8 weeks. Thereafter, participants will be cluster-randomized based on their office’s geographic location.

The interventions will start the following week and contain web-based educational sessions (Multimedia Appendix 1) and discussions about how to change behavior on an individual level to achieve a more sustainable and healthier lifestyle. Around 2-3 months after the final workshop, a follow-up meeting (workshop 7) will be held to discuss changes on an organizational level where participants will be encouraged to invite their workplace managers. All workshops will be in person at or near the participant’s workplace. The leaders of each workshop were trained staff from the researcher team at Mälardalen University with a background in nutrition and physiotherapy, and the same staff attended all workshops, except for the first baseline assessment where extra staff will help out with the logistics of data collection.

### A Framework for Behavior Change

The behavioral change wheel [32] serves as a framework for the novel multilevel (personal plus organizational) intervention, which is based on the framework of the COM-B model, including capability (C), opportunity (O), as well as motivation (M), which together have the capacity to support a change in behavior (B). Capability refers to the resources and skills necessary for the individual to adopt a healthy and sustainable lifestyle, for example, knowledge and culinary skills. Opportunity refers to organizational factors, policies, availability, and accessibility of resources supporting a healthy and sustainable lifestyle, for example, areas for physical activity and food options at work. Motivation refers to internal or personal factors that influence decision-making for adopting a healthy and sustainable lifestyle, such as a desire to improve one’s health, contribute to reducing local food waste, reduce one’s global footprint from food and transportation, and increase work productivity.

### Supportive Structures

The research team will facilitate behavior change among participants using the following behavior techniques: goal setting, self-monitoring, social support, framing and reframing, prompts and cues, action planning, information on behaviors and outcomes of behaviors, information about social and environmental consequences, and lastly, review of behavior goals and outcomes (Multimedia Appendix 2) [33]. The 5 web-based educational workshops will be grounded in the principles of the behavioral change techniques framing and reframing, prompts and cues, as well as information regarding social and environmental consequences. These sessions will actively engage participants in discussions and problem-solving, aimed at fostering action planning, and the study personnel will offer constructive feedback on behaviors and outcomes aligned with the individual goals set by the participants (for a description of the intervention functions, see Multimedia Appendix 3 [32,33]).

Participants will complete short, printed questionnaires at workshops 2, 4, and 6 to establish and assess individual dietary goals; monitor behavioral changes; evaluate goal fulfillment, self-efficacy, and outcome expectations; assess social support types and levels; and identify potential barriers and facilitators related to their dietary goals. Throughout the study, participants will complete these questionnaires for the purpose of self-monitoring and reviewing their progress toward achieving their behavioral goals and process evaluation [34]. The questionnaires take between 1 and 3 minutes to complete and will be administrated in paper format at the workshops; the questions are based on the behavior change wheel and social cognitive theory [32,35]. The collected data will be analyzed and summarized using quantitative methods to determine common perceptions of social factors that support behavioral change, identify facilitators, highlight individual experiences, and pinpoint perceived obstacles to implementing behavior changes.

A digital behavioral support package (guidelines, recipes, culinary skills tips, and food choices) will be provided to support adherence to the recommended advice about sustainable and healthy lifestyles, respectively. Recipes will be provided based on guidelines set by the Swedish Food Agency [36] for the control arm, and recipes contributing to a maximum of 0.5-1.5 kg of CO_2_e per portion developed by the Swedish World Wildlife Fund [37] and the Swedish consumer Agency [38] will be provided to the intervention arm. During the workshops, participants will be served lunch in line with the nutritional recommendations of the NNR [20], which is rich in plant-based food such as vegetables, legumes, seafood, and lacto-ovo-vegetarian options.

In addition, the individual-level education, instruction, and motivational factors for behavior change will be put in the context of environmental and organizational prerequisites for behavior change during workshops 1-4. This will be followed up during the fifth workshop in the intervention study when both arms will also be asked to use the research-supported Stanford Healthy Neighborhood Discovery Tool smartphone app (Discovery Tool) [39,40] to document contextual facilitators, barriers, or missing components in their work-related environments with regard to sustainable and healthy lifestyle. Barriers or problems in the workplaces’ physical, social, and organizational environment will be identified and solutions for change will be discussed and implemented during the final workshops for the intervention and control arm, respectively.

### The Intervention Arm: Sustainable Lifestyle

The participants in the sustainable lifestyle arm will be instructed to follow the current dietary and physical activity recommendations of the Food and Agriculture Organization of the United Nations, WHO, and NNR [6,20,22], as well as to adopt proenvironmental behaviors, defined as actions that minimize harm to the environment and combat climate change [41]. They will also be encouraged to increase active transportation to and from work, such as walking or biking [22]; use active transportation (walking) in and around the office; and break up prolonged sitting. The participants will be instructed to consume organically produced food to the highest possible degree and select food items that generate low amounts of greenhouse gas emissions by reducing their intake of red and processed meats.

### The Control Arm: Healthy Lifestyle

The participants in the healthy lifestyle arm will be instructed to follow the dietary recommendations by NNR [20]. In brief, they will be instructed to increase the intake of fruit and vegetable to at least 500 g per day and follow the recommended distribution of energy from macronutrients, that is, energy coming from fat, protein, and carbohydrates, as well as general recommendations about the “plate model,” the combination of food on the plate, to adhere to the recommended intake. Additionally, participants will be instructed to engage in physical activity corresponding to at least 150 to 300 minutes per week of moderate to vigorous intensity, according to the WHO and NNR [20,22]. Moderate-intensity activities include brisk walking, raking the yard, or biking to work, whereas vigorous activities comprise climbing stairs, jogging, or running [22,42].

### Questionnaire Data

We will use self-administrated web-based questionnaires (Multimedia Appendix 4) to assess quantitative data about study participants and lifestyle factors. The questionnaires are implemented in the online system Survey Generator by Alstra, and a unique and personal link to the questionnaire will be sent to each participant by email. The questionnaires are the same for both the intervention and control arm.

Dietary intake will be assessed using the food frequency questionnaire Meal-Q, developed and validated by us [28,29]. Meal-Q covers five domains of food consumption: (1) habitual intake of food items, dishes, and beverages including alcoholic beverages; (2) portion sizes; (3) eating behaviors; (4) meal patterns; and (5) supplements. Photos of (1) rice, potatoes, and pasta; (2) meat, chicken, fish, and vegetarian substitutes; and (3) vegetables are used to estimate portion sizes. For all other food items, a standard portion size will be used. Data from Meal-Q will be converted to a daily intake of approximately 50 nutrients by linkage to the National Food Composition Table [43] to generate the average intake of nutrients per person and day.

Also, life cycle assessment will be used to assess the carbon footprint of food consumption, covering the entire “cradle-to-grave” impact of products, and expressed as kilogram CO_2_e per kilogram of food products (kg CO_2_e/kg) [44]. In brief, we have collected data from life cycle assessment studies aiming at representing the average consumption pattern in Sweden, and each food item in the dietary questionnaire Meal-Q has been assigned a specific CO_2_e value and generates an average daily value of kg CO_2_e per person [45]. The dietary questionnaire also includes questions about the consumption of organic products, both with the type of food groups or products that are consumed as organic versions and the proportion of organic versions within each food group or product.

Physical activity will be assessed using Active-Q, developed and validated by us [30,31]. Active-Q covers five domains of activity: (1) daily occupational activity; (2) transportation to and from daily occupation; (3) leisure-time activity; (4) exercise; and (5) sleep. Participants report the type, duration, and frequency of various activities in each domain. Data from Active-Q are used to calculate the time spent at different activity levels, daily total metabolic equivalent tasks, and energy expenditure [30,46]. Also, Active-Q includes questions about active transportation, such as walking and biking, to and from daily occupations.

### Urinary Pesticide Residuals

To get an objective measure of the consumption of organic food, we will collect urine samples at baseline and at the end of the study to assess the excretion of pesticide residues. Participants will be asked to collect the first urine in the morning (between 6:00 and 8:00 AM) in 50 mL FALCON polypropylene containers and store it cold until the workshop later the same day (between 11.30 AM and 12:30 PM). Thereafter, all samples will be transported on ice to the Swedish Environmental Research Institute, where samples are to be stored at –80 ˚C until further analysis according to gold standard methods. The results will be calculated as μg pesticide/g creatinine to adjust for differences in urinary concentration.

### Anthropometrical Assessments

For participant characteristics and calculation of BMI, standardized procedures and equipment will be used to measure participant height (cm) by stadiometer Seca 213 (seca) and body weight (kg) by a bioelectrical impedance (BIA) Tanita BC-418 MA Multi-Frequency segmental body composition analyzer (Tanita) [47]. Data on body composition will be given to the participants as an incentive to join the study but will not be included in future data analysis.

### Photo and Narrative Documentation of Organizational Factors

The Stanford Healthy Neighborhood Discovery Tool smartphone app (Discovery Tool), based on the Our Voice citizen science framework [39], will be used during the fifth workshop of the sustainable and healthy intervention to document elements in the physical and organizational environment that either help or hinder employees’ attempts to be active and eat in healthy ways at work. Participants in the intervention arm will be instructed to take photos of barriers and facilitators for a sustainable lifestyle at or near the office, whereas the control arm will document barriers and facilitators for a healthy lifestyle, for 1 week. The app prompts the participants to add a text narrative explaining each photo. This makes it possible to highlight positive and negative aspects of the work environment, as well as suggest improvements and things that are missing. The participants can also leave a reaction to the photo in the form of positive or negative smiley faces. After taking photos, the participants upload the photos and accompanying Discovery Tool data to a secure server at Stanford University in anonymized form. From there, study personnel will access the data and download it to a local server at Mälardalen University. The participants in both intervention arms will discuss the photos at the sixth workshop of the intervention and create a list of 3-4 prioritized actions to be made until the follow-up workshop 7, which is 2-3 months after the intervention. This way, the participants can impact their everyday environment on an organizational level to facilitate a sustainable and healthy lifestyle, respectively. Discussions and comments during the workshops will be noted and transferred to electronic format. The analysis of photographs, audio narratives (voice-to-text), and additional comments are to be analyzed through the “By the People” Our Voice citizen science method [48-50], followed by a content analysis [51] to quantify and analyze the presence, meanings, relationships of certain words, themes, or concepts. One of the research team members will take the lead in coding, which will be reviewed and discussed by the remaining team members. Thus, the “Our Voice” method amplifies the voices and perspectives of participants throughout the scientific process [52], which ensures the trustworthiness and credibility of the results.

### Statistical Analyses

Data analysis will be performed according to the intention-to-treat principle [53], and all quantitative data will be analyzed using R statistics (R Foundation for Statistical Computing). Linear mixed-effects models [54] are planned for the analysis of intervention effects for numerical variables with a Gaussian distribution. These models are particularly suitable for repeated measures and can adeptly handle unbalanced groups. Linear mixed-effects incorporate both fixed and random effects, providing a robust method for accurately representing nonindependent data structures. For numerical variables with non-Gaussian distributions, the analysis will be conducted using generalized linear mixed-effects models. Generalized linear mixed-effects models are an extension of the generalized linear model in which the linear predictor contains random effects in addition to the usual fixed effects [54,55].

### Power Calculations and Randomization

Participants working in the targeted offices will be cluster-randomized based on the office’s geographic location by using a computer-generated random number generator in the statistical software R version 4.3.2 or later (Multimedia Appendix 5) [56]. Based on simulations derived from the pilot study CLEAR 2018 (unpublished data) using diet-related CO_2_e expressed as kg/day/person derived from Meal-Q, where we have an effect size of *η*^2^=0*.*11 for the interaction, a sample size of 40 observations in each arm at each assessment is indicated for 80% power. The estimated sample size was based on the interaction effect between control and intervention arms on CO_2_e in a linear mixed model maintaining a significance level of 5% from the CLEAR 2018 study. We assume a dropout rate of 10% to 15% after baseline assessment, and therefore, we will recruit 47 people to each arm, in order to have at least 40 at the end of the study. If the dropout rate in this study is less than 10%, no particular method will be used to account for attrition.

### Research Hypothesis

The study hypothesis is to investigate whether providing information about sustainable living encourages individuals to adopt more nutritious dietary habits and increase their levels of physical activity, as compared to receiving information solely centered around health-related recommendations for dietary intake and physical activity. The hypothesis will be tested among office workers to explore the workplace as an arena for health promotion.

### Primary Objective

The primary objectives are to examine the amount of change in the consumption of nutrients (fat, protein, carbohydrates, and fibers), more climate-friendly foods (increased intake of plant-based food and less dairy, red and processed meat, diet-related CO_2_e), more organic food, and increased practice of active transportation and related environmentally friendly behaviors. This will be evaluated by studying 1 variable at a time plus creating aggregated variables (the sums of all servings of fruits and vegetables expressed as servings per day), as well as creating a Sustainable Lifestyle Index, where each variable will be scored and summed to derive an overall score for each person [57], where higher scores indicate a more sustainable lifestyle. Finally, we will study the excretion of pesticide residues in urine as a validation of the consumption of organic food and explore if the excretion differs between the arms after the 8-week intervention.

### Secondary Objectives

Secondary objectives cover the evaluation of the participants’ workshop attendance rate; own goal setting; monitoring of behavior change (goal importance, self-efficacy, outcome expectations, and goal fulfillment); and supporting factors and obstacles regarding manager, colleagues, facilitating researchers, and family to study if there are any differences between the intervention and control arms (Multimedia Appendix 6). Also, data from the Discovery Tool app will be used to study if participants in the 2 arms identified different facilitators and barriers in the built, social, and organizational environment. Moreover, we aim to study if the intervention group reported a greater level of change in opinions and behaviors when it comes to environmental and climate aspects of their lifestyle, as compared to the control arm.

## Results

Funding for this study was granted by the Knowledge foundation (KKS) in October 2020 and approved by the Swedish Ethical Review Authority in June 2021, with an amendment approved in September 2022. The start of the study and recruitment of research team members were delayed due to the ongoing COVID-19 pandemic in 2021. However, we started informing potential study participants about the study during the spring of 2022 and were able to start the recruitment of participants and data collection in August 2022 (iteration 1), and the second round of data collection (iteration 2) started in March 2023. Iterations 1 and 2 will be referred to as the pilot phase of the SOFIA study.

Groups of 5 to 15 participants were included in the intervention and control arms, respectively, during each iteration of the data collection, and the data collection will continue in the coming years. As of June 2024, a total of 37 participants have been recruited, of which 21 were included in the intervention arm and 16 in the control arm. The dropout after randomization was 2 in the intervention arm and 2 in the control arm, resulting in data from 19 participants in the intervention arm and 14 in the control arm, and a dropout rate of less than 10%. A majority of the participants are female (24/33, 73%) and are working in company B (22/33, 67%). The results of the pilot phase are expected to be published in 2024 or 2025.

## Discussion

### Principal Findings

This study aims to increase the understanding of the effectiveness of promoting a sustainable lifestyle to motivate behavioral change toward more nutritious food habits and increased physical activity, as compared to providing information based exclusively on health-related arguments in line with recommendations for food habits and physical activity. By targeting motivation, capabilities, and opportunities based on the behavioral change wheel model [58], we intend to increase the effect of the intervention in this specific context; moreover, it improves the understanding of behaviors in relation to the workplace environment [32]. A potential primary finding of this study would be that the intervention arm that received information about a sustainable lifestyle improved their food habits and physical activity level equally, or more, as compared to the control arm receiving information about health-related recommendations. Additionally, it is anticipated that the study will show that a shift to a sustainable lifestyle will lower diet-related CO_2_e emissions and reduce the excretion of urinary residuals of pesticides to a larger degree in the intervention arm than in the control arm.

A novel feature of this study is the use of the workplace as an arena for health promotion, and in particular office workers, a group known to be sedentary at work [59]. A substantial proportion of the adult population spends a significant amount of time at work each day, and the advantage of an office-based intervention is that many individuals can participate simultaneously at work [23,60]. Moreover, from the employee’s perspective, these types of interventions are convenient and easily accessible and allow employees to interact and support each other in their efforts to undertake behavior changes [61]. Full-time employees spend up to 60% of their waking hours at work [62] and typically return repeatedly to the same location, providing a significant opportunity to deliver health interventions. Recent review articles show that multicomponent workplace dietary interventions are effective but also an unutilized area to positively influence dietary intake and health outcomes among office workers [63]. However, the intervention needs to be tailored to the workplace and use behavioral and cognitive strategies to be successful [64,65]

In addition, we focus on the individual’s behavior change as well as features on an organizational level to facilitate or hinder a sustainable or healthy lifestyle at work. By implementing a citizen science methodology within the trial, the participants (citizen scientists) collect data using the app, the Discovery Tool; are involved in analyzing the data and formulating a list of actions; and contacts managers to bring about changes in their workplace.

### Comparison to Prior Work

We are not aware of any similar work, but a recent randomized controlled trial was conducted to assess the effects of an app-based intervention to improve the dietary intake of people with type 2 diabetes [66]. The intervention lasted for 12 weeks, with each week covering a new diet-related topic, with emphasis on the recommended intake of fruits, vegetables, legumes, and whole-grain products and reduction of red and processed meat. Although the primary aim was not to reduce diet-related greenhouse gas emissions, nor did the intervention study include information about carbon footprint, it was hypothesized that behavior changes toward more healthy food choices, would also result in lower emissions of CO_2_e expressed as kg/day/person. However, ad hoc analyses of CO_2_e did not show any differences between the intervention and the control arm.

### Strengths and Limitations

A strength of the study is the low dropout rate, indicating that the participants found the study engaging and motivating. However, the pilot phase of the intervention study is relatively small and targets office workers at progressive companies, of which most are well educated and interested in healthy behaviors. Thus, it potentially leaves limited room for improvement over the 8-week intervention. Also, we will collect data related to a large number of exposures that may introduce the risk of chance findings.

### Future Directions

As of the summer of 2024, the data collection of the pilot phase of the SOFIA study has ended, and the analysis of data is ongoing. A continuation of the recruitment process is planned for the coming years until the target number of participants is met. For now, this study protocol may serve as a framework to guide both scholarly research and the implementation of lifestyle interventions in office contexts. We anticipate that the company partners, where the SOFIA intervention was conducted, may implement aspects of the study to bring about change in their organizations. Additionally, future studies should include more diverse groups of employees, for example, from the public sector as well as blue-collar workers

### Conclusions

In conclusion, given the ongoing climate change, negative environmental effects, and the global epidemic of metabolic diseases, a sustainable lifestyle among office workers holds an important potential to counteract this trend. There is an urgent unmet need to test the impact of a sustainable lifestyle intervention on food intake, physical activity, and environmental and climate-friendly behaviors. Thus, this study protocol responds to a societal need, by covering behavior changes of importance for successful implementation of sustainable lifestyle habits in an office setting.

## Appendix

Multimedia Appendix 1 Themes for workshops 1-5 for the experimental and control arms.

Multimedia Appendix 2 Program theory.

Multimedia Appendix 3 Intervention functions.

Multimedia Appendix 4 Screenshot showing portion sizes of food in the web-based dietary questionnaire.

Multimedia Appendix 5 R-syntax for randomization.

Multimedia Appendix 6 Questionnaire for goal setting and monitoring of behavior change translated to English.

Multimedia Appendix 7 Approved grant proposal.

## Abbreviations

CO2e: CO2 equivalents
NCD: noncommunicable disease
NNR: Nordic Nutritional Recommendations
SOFCO: Concepts for the Sustainable Office of the Future
SOFIA: Sustainable Office Intervention
WHO: World Health Organization

## Data Availability

The data sets generated or analyzed during this study are available from the corresponding author on reasonable request.
