# Supplementary material for: Sustainable Lifestyle Among Office Workers (the SOFIA Study): Protocol for a Cluster Randomized Controlled Trial
Source: JMIR Res Protoc. 2024 Jul 31;13:e57777. doi: 10.2196/57777 (PMC11325103; doi:10.2196/57777)
Supplement: Multimedia Appendix 1 [file resprot_v13i1e57777_app1.docx]

Appendix 1

The themes for the educational workshops in the SOFIA study divided by the intervention group (sustainable lifestyle) and the control group (healthy lifestyle), respectivly.

| **Workshop** | **Sustainable lifestyle** | **Healthy lifestyle** |
| --- | --- | --- |
| 1. | - Introduction to a sustainable lifestyle. - Global food consumption and disease burden - The impact of food production and consumption on climate and environmental issues. - Dietary guidelines with focus on recommended intake of red meat and processed meat as well as on fruits and vegetables, and fiber - The recommended proportion of energy form different macro nutrients (fat, protein, and carbohydrates). - The role of energy balance for maintaining a normal body weight. - Win-win situation between climate and healthy aspects when eating plant-based food. | - Introduction to a healthy lifestyle. - Global food consumption and disease burden - Dietary guidelines with focus on recommended intake of red meat and processed meat as well as on fruits and vegetables, and fiber |
| 2. | - The climate and environmental impact of organic farming vs. conventional farming. - Consumption of organic products. - How organic products are labeled on a national and European level. | - The recommended proportion of energy form different macro nutrients (fat, protein, and carbohydrates). - The role of energy balance for maintaining a normal body weight. - The pedagogical model “the plate model” to guide the proportion of various foods on the plates. |
| 3. | - The carbon footprint from food production, transportation, and consumption. - Be aware of food waste. | - Being a conscious consumer. - How to read a table of content on food products. - Different types of carbohydrates - The sugar and fiber content in different food products. |
| 4. | - The trade-off between healthy foods on one hand and their climate and environmental impact on the other hand. - How to use the food guide on climate and environmental impact from WWF. - Protein from plant and animal-based foods. - Recommended intake of vitamins and minerals | - Be aware of food waste. - How products are labeled including faire trade, sustainable stocks, key hold (healthy foods) on a national and European level. |
| 5. | - Being a conscious consumer. - How to read a table of content on food products. - Different types of carbohydrates. - The sugar and fiber content in different food products. - Introduction to citizen science and the use of the Discovery Tool to document facilitators and barriers for a sustainable lifestyle at work. | - Recommended intake of vitamins and minerals. - Food intake vs. supplements - Introduction to citizen science and the use of the Discovery Tool to document facilitators and barriers for a healthy lifestyle at work. |
| 6. | - Being a citizen scientist and analyzing photos of facilitators and barriers for a sustainable lifestyle at work. - List 3-4 prioritized barriers at the workplace and advocate for change on an organizational level. | - Being a citizen scientist and analyzing photos of facilitators and barriers for a healthy lifestyle at work. - List 3-4 prioritized barriers at the workplace and advocate for change on an organizational level. |
