# Supplementary material for: Sustainable Lifestyle Among Office Workers (the SOFIA Study): Protocol for a Cluster Randomized Controlled Trial
Source: JMIR Res Protoc. 2024 Jul 31;13:e57777. doi: 10.2196/57777 (PMC11325103; doi:10.2196/57777)
Supplement: Multimedia Appendix 2 [file resprot_v13i1e57777_app2.pdf]

Behavior change Wheel (BCW)

Behavior techniques taxonomy by Michie et al., 2009

Problems identified

- Low adherence to NNR/WHO.
- High anxiety and low self-efficacy towards combating climate change.
- High rates of greenhouse gas emissions from food and transports
- Lack of knowledge regarding the climate impact of food.
- Low motivation for active transportation to work.
- Social norms and negative attitudes towards vegetarian and vegan food.
- Fear of not getting enough protein from vegan food.

Intervention (Inputs, assumptions and mediators of change)

Workshops and education | **Behavioral support package** | **Test and taste new foods** | **Citizen science**

Increased knowledge, self-efficacy and motivation via practical actions to combat climate change in social networks at work that facilitate changes in norms, attitudes, and behaviors.

- Goal setting
- Self-monitoring
- Framing and reframing
- Social support
- Action planing
- Review of behavioral goals
- Information about social and environmental consequences
- Prompts and cues
- Feedback on behaviors and outcomes
- Information on behaviors and outcome of behaviors

Workshops 1 to 6

Critical assumptions:
- Behavioral support package is easy to use and reach all participants.
- Participants gain trust in facilitators
- Sustainable foods becomes accepted
- The workplace provide social support
- Messages are perceived as relevant

Context
- Ceiling effect at baseline
- Office environment
- Time/season of the year
- Access to healthy and sustainable foods
- Working from home

Stakeholders
- Company CEO
- HR/managers
- Restaurant managers

Intermediate outcomes

- Improved knowledge.
- Increased self-efficacy towards consuming healthy and sustainable food and to combat climate change.
- Changes in outcome expectations.
- Improved action planning
- Autonomic behaviors.
- Improved awareness of the climate impact of food.
- Enhanced social support at work.

Final outcomes

Increased intake of:
- Fruits and vegetables
- Fiber and nutrients

Increased:
- Active transportation
- Physical activity

Decreased CO2e emissions from food.

Changes in urinary pesticide levels

What changes have been implemented in the workplace 2-3 months post-intervention? Based on the Discovery tool workshop.

Sources of errors:
- Metabolites in urinary pesticide levels are sensitive to sudden changes
- self-reported data
- Allocation concealment fail
- Private life and family situation
