# Supplementary material for: Sustainable Lifestyle Among Office Workers (the SOFIA Study): Protocol for a Cluster Randomized Controlled Trial
Source: JMIR Res Protoc. 2024 Jul 31;13:e57777. doi: 10.2196/57777 (PMC11325103; doi:10.2196/57777)
Supplement: Multimedia Appendix 3 [file resprot_v13i1e57777_app3.docx]

**Intervention functions and support content in the SOFIA-study**

| **Capability (C) Opportunity (O)**  **Motivation (M)**  **(Michie et al., 2011)** | **COM-B intervention functions** | **Facilitators for behavior change** | **Implementation support (Michie et al., 2013)** | **Description of the implementation support** | **Workshop 1-6 (7)** |
| --- | --- | --- | --- | --- | --- |
| M | Incentivization | Participants need to set a diet-related goal and have high outcome expectations and self-efficacy toward goal achievement. | Goal setting | Supporting participants to choose a specific, relevant, and achievable, time-based goal. | 2 |
| M | Incentivization and restriction | Participants needs to fill-out the follow-up questionnaire regarding the diet-related goal. | Self-monitoring | Feedback on goals and review of behavioral goals from the facilitating researchers. | 4 and 6 |
| O | Environmental restructuring | Create a collegial support structure at work. | Social support | Workshops and education in groups, using the workplace as a social arena for health promotion. | 1 to 6 |
| C, M | Education | Participants needs to adopt information and put it into practice. | Framing and reframing | Provision of lunch at workshops (including less/no animal products). Moreover, information about that a sustainable lifestyle is also a healthy lifestyle. | 1 to 6 |
| C | Education | Participants needs to choose products marked with relevant food labels. | Prompts and cues | Information and communication on food labels (Swedish Keyhole symbol and KRAV). | 2 |
| C, M | Education and incentivization | Participants needs to adopt information and put it into practice. | Action planning | Information and communication regarding strategies such as: grocery planning and active commuting strategies. | 1-6 |
| C, M | Education | Participants needs to fill-out the questionnaire and participate in workshops. | Information on behaviors and outcomes of behaviors | Discussions of healthy sustainable behaviors at workshops and how they affect health and the environment. | 2, 3 and 4 |
| C, M | Education | Participants needs to adopt information and put it into practice, to e.g., reduce red meat consumption. | Information about social and environmental consequences. | Education, relevant examples of the environmental and health consequents of e.g., high meat consumption. | 1-6 |
| M | Incentivization | Participants needs to fill-out the questionnaire and participate in workshops. | Review of behavior goals and outcomes | Feedback on goals and review of behavioral goals based on questionnaires and during workshops. | 4 and 6 |
| O | Education and enablement | Participants needs visit the Digital support package webpage, moreover, adopt the information and put it into practice. | Digital behavioral support package | Provision of guidelines, recipes, culinary skills tips, and food choices. | 1 to 6 |
| C, M | Modelling and enablement | Participants need to participate in workshops and eat the food that is provided. | Provision of lunch at workshops. | Test and taste legumes, seafood, and lacto-ovo-vegetarian options provided at the workshops. | 1 to 6 |
| O | Environmental restructuring | Participants need to take pictures of the workplace and discuss the collected material. | Citizen science (Discovery Tool) | Identification of facilitators, barriers, or missing components in the built, social, and organizational environment at work. | 5, 6 and 7 |
