# Supplementary figures and images for: Sustainable Lifestyle Among Office Workers (the SOFIA Study): Protocol for a Cluster Randomized Controlled Trial
Source: JMIR Res Protoc. 2024 Jul 31;13:e57777. doi: 10.2196/57777 (PMC11325103; doi:10.2196/57777)

Appendix 4.

Screen dump of the web-based dietary assessment showing examples of portion sizes.


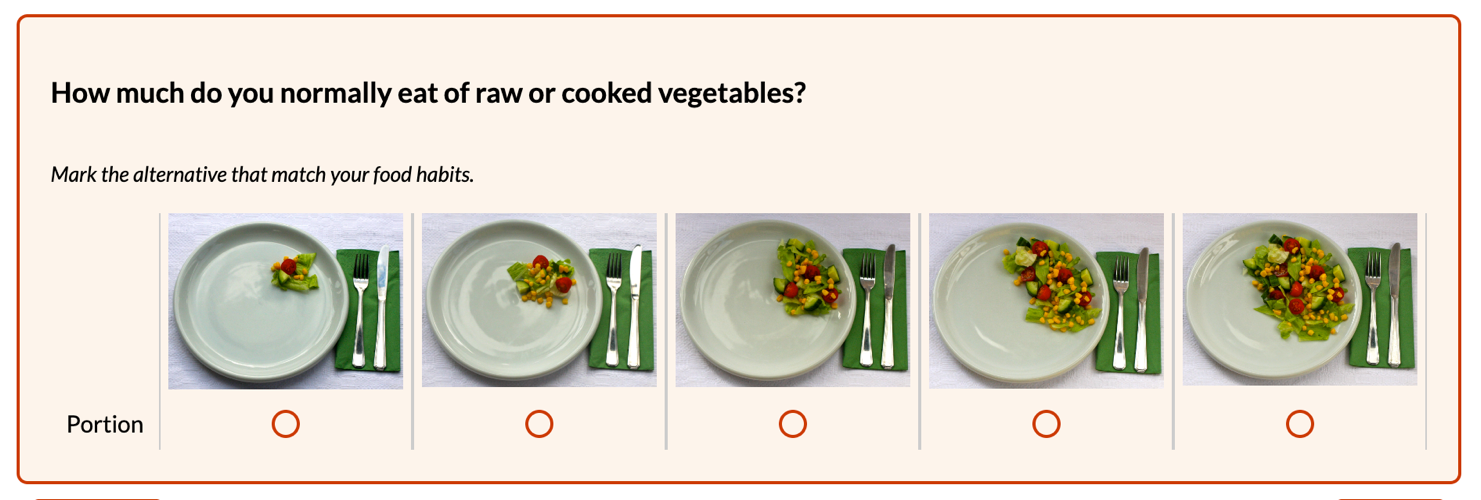

Supplement: Multimedia Appendix 4 [file resprot_v13i1e57777_app4.docx]
