# Supplementary material for: Sustainable Lifestyle Among Office Workers (the SOFIA Study): Protocol for a Cluster Randomized Controlled Trial
Source: JMIR Res Protoc. 2024 Jul 31;13:e57777. doi: 10.2196/57777 (PMC11325103; doi:10.2196/57777)
Supplement: Multimedia Appendix 5 [file resprot_v13i1e57777_app5.docx]

# File: randomization.R

# Date: 2022-10-07

# Author: Annika Tillander and Oskar Halling Ullberg

# Purpose: Randomize the two groups into the different educations: sustainable lifestyle (A) or healthy lifestyle (B)

# Here we can replace A and B if you want or define them

# A is group (“company cluster”)

# B is group (“company cluster”)

Groups <- c("A", "B")

# Set the seed of Râ..s random number generator to get same result each time program/code is run, using the date when the program was written makes most sense.

set.seed(20221007)

# Selected for education 1

edu1 <- sample(Groups, size = 1)

edu1

# Selected for education 2

edu2 <- Groups[Groups!=edu1]

edu2
