# Supplementary material for: Sustainable Lifestyle Among Office Workers (the SOFIA Study): Protocol for a Cluster Randomized Controlled Trial
Source: JMIR Res Protoc. 2024 Jul 31;13:e57777. doi: 10.2196/57777 (PMC11325103; doi:10.2196/57777)
Supplement: Multimedia Appendix 6 [file resprot_v13i1e57777_app6.docx]

1. **Questionnaire about goals, expectations, and motivation regarding changes in dietary habits (At workshop 2).**

- Briefly describe your goal:

**Encircle the number that best corresponds to the question:**

- How important is it for you to achieve your goal within the next two weeks?

0 1 2 3 4 5 6 7 8 9 10

Not important Very important

- How confident are you in your ability to achieve your goal within the next two weeks?

0 1 2 3 4 5 6 7 8 9 10

Uncertain Very confident

- (Only Sustainable lifestyle) To what extent do you believe your choices of food products impact the planet?
- (Only Healthy lifestyle) To what extent do you believe your choices of food products affect your health?

0 1 2 3 4 5 6 7 8 9 10

No impact Significant impact

**2. Questionnaire about goals, expectations, and motivation regarding changes in dietary habits (At workshop 4).**

- Briefly describe your goal:

**Encircle the number that best corresponds to the question:**

- To what extent have you achieved your goal on a scale of 0-10 in the past two weeks?

0 1 2 3 4 5 6 7 8 9 10

Not at all Completely fulfilled

- How important is it for you to achieve your goal within the next two weeks?

0 1 2 3 4 5 6 7 8 9 10

Not important Very important

- How confident are you in your ability to achieve your goal within the next two weeks?

0 1 2 3 4 5 6 7 8 9 10

Uncertain Very confident

- On a scale of 0-10, how do you perceive your support from... in changing your behavior and reaching your goal in the past two weeks?

**From family:** Bad 0 1 2 3 4 5 6 7 8 9 10 Very good

**From colleagues:** Bad 0 1 2 3 4 5 6 7 8 9 10 Very good

**From manager/team leader:** Bad 0 1 2 3 4 5 6 7 8 9 10 Very good

**From the researchers:** Bad 0 1 2 3 4 5 6 7 8 9 10 Very good

- What is preventing you from reaching your goal?
- What facilitates your ability to reach your goal?

**3. Questionnaire about goals, expectations, and motivation regarding changes in dietary habits (At workshop 6).**

- Briefly describe your goal:

**Encircle the number that best corresponds to the question:**

- To what extent have you achieved your goal on a scale of 0-10 in the past two weeks?

0 1 2 3 4 5 6 7 8 9 10

Not at all Completely fulfilled

- On a scale of 0-10, how do you perceive your support from... in changing your behavior and reaching your goal in the past two weeks?

**From family:** Bad 0 1 2 3 4 5 6 7 8 9 10 Very good

**From colleagues:** Bad 0 1 2 3 4 5 6 7 8 9 10 Very good

**From manager/team leader:** Bad 0 1 2 3 4 5 6 7 8 9 10 Very good

**From the researchers:** Bad 0 1 2 3 4 5 6 7 8 9 10 Very good
