# Supplementary material for: Sustainable Lifestyle Among Office Workers (the SOFIA Study): Protocol for a Cluster Randomized Controlled Trial
Source: JMIR Res Protoc. 2024 Jul 31;13:e57777. doi: 10.2196/57777 (PMC11325103; doi:10.2196/57777)
Supplement: Multimedia Appendix 7 [file resprot_v13i1e57777_app7.pdf]

DeepL

Subscribe to DeepL Pro to translate larger documents.
Visit www.DeepL.com/pro for more information.

PART 1

From: KK Foundation <info@kks.se>
Sent: June 17, 2020 11:28 am
To: Katarina Bälter <katarina.balter@mdh.se>
Subject: Decision on the Synergy 19 call for applications

KK·stiftelsen

Date	Record number
2020-06-17	20200068

Mälardalen University

Principal Paul Pettersson

Box 883 721 23 Västerås

Decision on the application Concept for the sustainable office of the future

The project application has been assessed by the Knowledge Foundation's assessment group for Synergy 19 according to the criteria stated in the call, based on the review of the written application and hearing. A statement from international experts has been obtained as an additional basis for the assessment.

At its meeting on June 16, 2020, the Board of the Knowledge Foundation has decided in accordance with the recommendation of the CEO and the assessment group to instruct the CEO of the Knowledge Foundation to initiate negotiations and conclude an agreement with the university regarding the financing of your above application. Please note that the project is not granted funding until the agreement is completed and signed by both parties.

Before an agreement is concluded, the conditions in the assessment group's justification must be met. These must be received by the Knowledge Foundation no later than September 1, 2020, including a letter of intent from co-producing companies.

We ask your communications officer or equivalent to contact the Knowledge Foundation's communications officer Petter Zirath, petter.zirath@kks.se, tel: 0737-12 81 35, for a discussion on how to jointly communicate the project.

Below is the review team's rationale for its decision. Scientific

quality
There is a good understanding of current research and the scientific basis of the project; and

the group has a relevant scientific competence. The proposed research addresses important and current societal problems. However, the application uses concepts such as "green office work", which are not clearly defined. Furthermore, there are parallel objectives linked to human health and climate sustainability. For example, the objective for subproject one is described as: "...encourage and nudge a sustainable lifestyle in an office context in order to improve public health and combat climate change". There is a clear duality here that can be perceived as focusing on behavioral changes rather than on the production of new knowledge. Although the scientific description is clear and linked to the state-of-the-art, the project description would have benefited from being less anecdotal and normative.

Benefits for business
The eight companies that are project partners should benefit significantly from the project and its results. The composition of the companies is interesting. However, the co-production and the roles of the companies in the project could have been described more clearly and in more detail, especially at sub-project level. In some cases the description is too anecdotal (see for example chapter 5.2.5).

Expected results and impacts
The interdisciplinary approach is highly relevant to the synergy and should be able to contribute to the university's development and profiling. The group has sufficient competence and should, together with the companies, be able to achieve the goals and take steps in the direction of the development in the work environment/workplace environment sought in the application. The publication plans are clear and well thought out.

Implementation
The applicants have the skills and capabilities to meet the objectives. However, the actual contribution of the companies needs to be described in more detail. Furthermore, the list of key persons' contributions needs to be adjusted. In the application, four persons currently have only five percent allocated to the project and one person seven percent. This seems to be low. In addition, two people have zero percent (!) allocated to the project, which must surely be a misprint. Another question is the appropriateness of people from the Steering Committee also being on the Scientific Advisory Board. These issues need to be clarified before a possible grant is awarded.

Summary
The project is important and the applicant constellation has good competence and opportunities to achieve the objectives. Overall, the assessment team considers that the application should be granted, provided that the following conditions are met:
• A clearer and more detailed description of the motivation and commitment of the companies is produced for all sub-projects and for the synergy.
• Concepts in the application such as "green office work", etc. need to be defined.
• The list of key persons needs to be corrected and clarified regarding their respective involvement in the project.
• The relationship between the Steering Committee and the Scientific Advisory Board needs to be explained and justified.

With kind regards
FOUNDATION FOR KNOWLEDGE AND SKILLS DEVELOPMENT

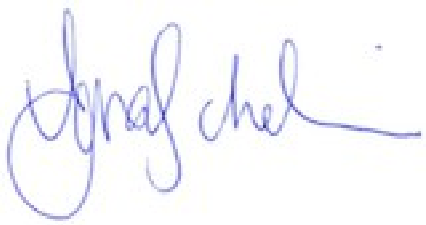

Eva Schelin
Executive Director

Copy:
Katarina Bälter
Registrar

FOUNDATION FOR KNOWLEDGE AND SKILLS DEVELOPMENT
Birger Jarlsgatan 16, 114 34 Stockholm, Sweden
Org.nr 802400-4213 www.kks.se

PART 2

The Foundation's financing of the Project amounts to SEK 11,963,982. OH will be added, see point 4 below.

3 Reporting plan

The university fully understands that the Foundation, in its decision on support, considered it particularly important that reporting takes place as agreed. Reporting is an essential part of the Foundation's ability to monitor and provide relevant support. The HEI shall report to the Foundation on the Project as described below.

Reporting is done via the Foundation's portal, see www.kks.se.

Report name	Latest KK Foundation available	Listing
Interim report	2022-11-30	incl. economic rationale
Final report	2024-11-30	including final financial report

The Foundation is entitled to terminate the project support and demand repayment of all or part of the support amount if the report is not submitted on time or does not meet the requirements set out in the report template.

During the project period, the Foundation has the right to request a dialogue with the project manager in order to familiarize itself with the progress of the Project, e.g. in terms of activities and results achieved in relation to the project description. Such a dialog should normally take place at the University. If the Foundation so requests, a representative from the participating company shall participate.

4 Payment plan

Payment of grants will be made according to the payment schedule below. Payments may be stopped if the Foundation has reason to believe that the project description is not being followed.

Payment info (pgfbg)	5201-5864
Payment reference ("Mark the stub with")	- - 2 zm

Date	Amount (SEK)	Listing
2020-12-15	1.793.097	
2021-06-15	1.793.097	
2021-12-15	1.793.097	
2022-06-15	1.793.097	
2022-12-15	1.793.097	
2023-06-15	1.793.097	
2023-12-15	1.793.097	
2024-06-15	1.793.099	
Total:	14.344.778	The amount includes OH by 20%.

It is noted that funds from the Foundation may not be used for purposes other than the Project.

5.10 Changing conditions

All amendments and additions to this Agreement shall be made in writing in order to be valid.

If conditions change significantly and the Foundation and the University cannot agree on the content of a new agreement, the Foundation may terminate this agreement with immediate effect. What has been paid to the University shall in such cases be repaid to the Foundation if negotiations between the parties do not lead to a different decision by the Foundation.

5.11 Non-transferability

The rights and obligations under this agreement cannot be transferred without the Foundation's written consent.

5.12 Conditions for the entry into force of the agreement

The agreement will enter into force once it has been received by the Foundation with the required signatures by September 28, 2020 and signed by the CEO of the Foundation.

6 Signatures of parties

Stockholm, '''p| FOR

Knowledge and skills development foundation

Eva Åkhelin
Verkställande direktör
Executive Director

Västerås on I '' JU

Mälardalen University

PaulPettersson
Principal

Anna Letterstål
Project owner

Katarina Bälter
Project Manager
